# Cutaneous metastases of embryonal rhabdomyosarcoma in a black African child: the role of dermoscopy in reducing time to and cost of diagnosis

**DOI:** 10.3332/ecancer.2021.1304

**Published:** 2021-10-12

**Authors:** Nkechi Enechukwu, Ezejiofor Ogochukwu Ifeanyi, Gabriel Olabiyi Ogun, Adebola Olufunmilayo Ogunbiyi

**Affiliations:** 1 Dermatology Unit, Department of Medicine, Nnamdi Azikiwe University/Nnamdi Azikiwe University Teaching Hospital Nnewi, Nnewi 435101, Nigeria; 2 Department of Pathology, College of Medicine, University of Ibadan, Ibadan 200212, Nigeria; 3 Department of Medicine, College of Medicine, University of Ibadan, Ibadan 200212, Nigeria

**Keywords:** dermoscopy, cutaneous metastases, embryonal rhabdomyosarcoma, Africa

## Abstract

Cutaneous metastases are an uncommon feature of solid organ malignancies. The cost of multiple investigations and prolonged processing time of biopsies may lead to diagnostic delays especially in resource limited practice settings. Dermoscopy can provide useful clues and has been found to be useful in the diagnosis of cutaneous metastases. Dermoscopic findings of skin lesions may limit unnecessary investigations and shorten time to diagnosis. There are limited data on dermoscopic features of embryonal rhabdomyosarcoma in the literature. We report dermoscopic features of cutaneous metastasis of embryonal rhabdomyosarcoma seen in a black African child.

## Introduction

Dermoscopy is a cheap, non-invasive procedure that allows in vivo assessment of structures and colours in the epidermis and papillary dermis not visible to the unaided eye [1]. Dermoscopy serves as a quick and accessible screening tool especially in resource limited practice settings where lack of funds may lead to a delay in diagnosis.

Embryonal rhabdomyosarcoma (RMS) is the commonest form of RMS observed in children. Although primary cutaneous disease may occur, cutaneous metastases are rare in children. Clinical distinction between these two is challenging and prognosis is poor in the latter. There are sporadic reports of cutaneous metastases of RMS in the literature [2–5]. However, no documentation of the dermoscopic features was made in these reports. As in all cutaneous malignancies, diagnosis may be facilitated by a high index of suspicion coupled with dermoscopic patterns consistent with cutaneous metastases. Data on the dermoscopic features of cutaneous metastases of organ tumours is limited [6]. We report the dermoscopic findings of cutaneous metastases from embryonal rhabdomyosarcoma in a Nigerian child.

## Case report

A 12-year-old girl presented with 1 month history of multiple painless, pruritic skin eruptions on her lower abdomen, vulvar and left lower extremities. She gave a history of progressive oedema and multiple indistinct swellings over her left lower limbs that occurred 7 months before the rash started. There was progressive abdominal swelling, marked weight loss, recurrent fever and breathlessness. Prior to the dermatology consultation, she had been investigated for an intra-abdominal malignancy of uncertain site.

**Figure 1.**
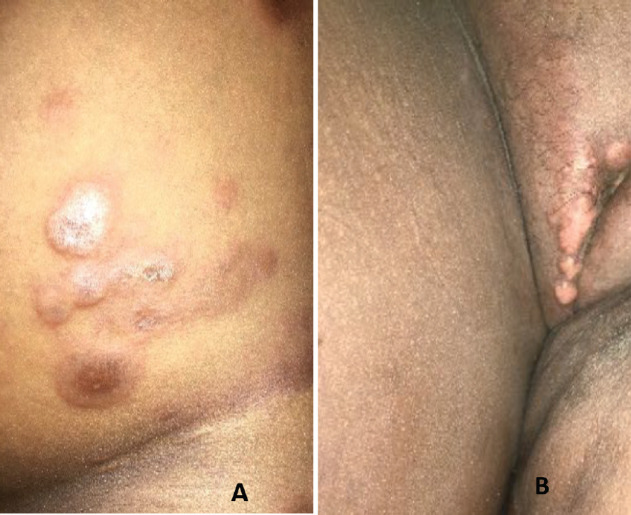
Multiple flesh coloured plaques on the (a): lower trunk thighs and (b): vulva.

There was no history of haematuria, urinary tract symptoms, vaginal mass or bleeding. Physical examination revealed abdominal distention with positive fluid thrill, multiple skin coloured nodules with background erythema on the lower abdomen, left thigh and labia majora and erythematous nodules with central hyperpigmentation and scales on the lower abdomen (Figure 1a and b).

Blood film was negative for microfilaria, abdominopelvic CT scan and ultrasonography showed massive ascites, left pleural effusion, multiple retroperitoneal and left inguinal lymphadenopathy, normal uterus and poorly appreciated ovaries (due to small size at the age and distortion from ascites). The plain radiograph of the left lower limb showed soft tissue swelling with no affectation of the underlying bone whilst CT Angiography showed gross edematous swelling of the left lower limb without any evidence of deep venous thrombosis or atheromatous vascular disease. Ascitic fluid cytology showed a hypercellular smear showing malignant cells with signet ring morphology with irregular nuclear margins, vesicular nuclei with prominent nucleoli in some areas and hyperchromatic nuclei in others.

**Figure 2.**
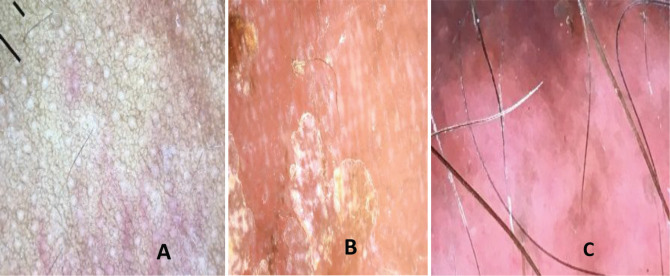
Dermoscopy showing multiple curvilinear erythematous lines, barely perceptible polymorphic vessels, whitish streaks, distorted pigment network and yellowish white scales.

Dermoscopy showed multiple curvilinear erythematous areas, barely perceptible polymorphic vessels, whitish streaks, distorted pigment network, and fine white scales (Figure 2a–c). A diagnosis of cutaneous metastases from an internal malignancy possibly pelvic malignancy was made.

**Figure 3.**
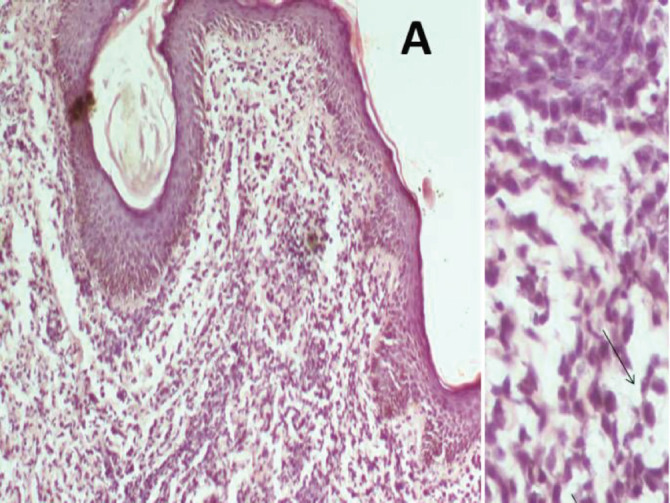
Histopathology (H&E) shows discohesive nests and sheets of malignant small round blue cells having hyperchromatic to vesicular nuclei, prominent nucleoli and scant to moderate pale eosinophilic cytoplasm.

**Figure 4.**
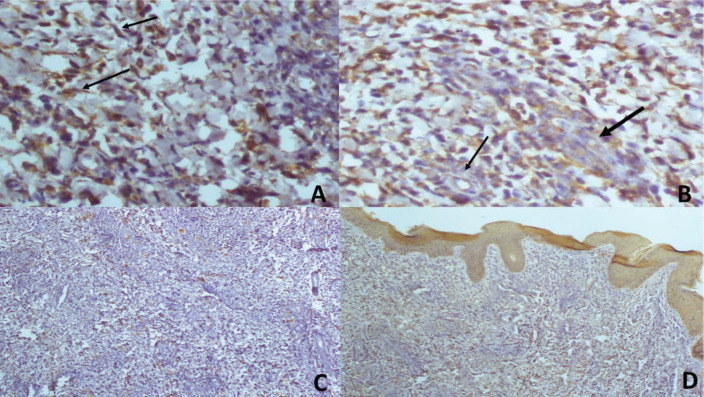
(a and b): (×200) The strap cells positive for desmin. (a): The typical strap cells (arrows). (b): Perivascular cuffing (arrows) with invasion of the vessel highlighted with the thicker arrow. (c): (×100) S-100 positivity only in the nerve twigs. (d): (×100) AE1/AE3 positivity in the overlying epithelium which serves as internal positive control and positivity in the prominent vascular channels in the section.

Histopathology of skin tissue obtained from the Right labia majora showed dermis infiltrated by sheet of hyperchromatic tumour cell in a prominent myxoid stroma (Figure 3a (×100)) and typical strap cells with eosinophilic cytoplasm (thin arrows) (Figure 3b (×400)). Immunohistochemistry of the lesional tumour cells was positive for Desmin, illustrating the myogenic origin of the strap cells and negative to cytokeratin and S100 (Figure 4a–d). A histologic diagnosis of embryonal rhabdomyosarcoma was made. The vaginal wall was considered as the possible site of the primary tumour.

The patient was subsequently referred to an oncology centre in another institution but unfortunately, she passed on before further investigations followed by chemotherapy and radiotherapy could be commenced.

## Discussion

Cutaneous metastases are an uncommon manifestation of internal malignancies and portend a poor prognosis [7, 8]. Its incidence ranges from 0.6% to 10% in some case series [9–10]. Dermoscopic clues of cutaneous malignancies do not differ from that of cutaneous metastases and include ulceration, white clues (white lines) or keratin clues in raised lesions (dermatoscopic white circles, dermatoscopic white structureless areas or surface keratin) and characteristic vascular morphologies [6, 7]. Dermoscopy of cutaneous lesions in patients with suspected malignancies can therefore be clarifying. With dermoscopic examination, cutaneous metastases often appear as skin coloured nodules or plaques as was seen in the index patient [6–8]. Others may present as erythematous papules, pink nodules and hyperpigmented nodules [2, 3, 6–10] Different vascular patterns including serpentine, dotted, glomeruloid, irregular hair pin-like or polymorphous vessels may be seen as in the index patient [6, 7]. The vascular morphology may be masked in darker skin phenotypes as in the index patient due to pigmentation. Consequently, in the absence of vascular patterns, other findings like ulcerations white lines, ulcerations may serve as useful clues [6]. Although there are currently no documented specific dermoscopic findings in cutaneous metastases of embryonal rhabdomyosarcoma, the curvilinear erythematous areas are a unique finding in the index patient. However, more case reports are needed to further validate this and to ascertain its significance. In skin of colour, distorted pigment network as seen in the index patient although non-specific is often associated with fibrosis which is a frequent finding in malignant skin lesions. There are currently no documented dermoscopic features of RMS in any racial group for comparison.

## Conclusion

Dermoscopy could serve as a useful tool in early diagnosis of cutaneous metastases of RMS in resource limited practice settings. This may ultimately limit unnecessary investigations and shorten the time to definitive diagnosis. White lines, polymorphic vessels and curvilinear erythematous structures are dermoscopic features of cutaneous metastases of embryonal RMS in a black African child. Vascular morphologies may be difficult to visualise and pigment network may be distorted.

## Conflicts of interest statement

The authors have no conflicts of interest to disclose.

## Funding declaration

The authors declare that they have not received fund(s) or financial support for the authorship and/or publication of this article.
